# Clinical effects of targeted forefoot and rearfoot training on dynamic balance, postural stability, gait biomechanics, and joint function in individuals with chronic ankle instability: study protocol for a prospective randomized controlled trial

**DOI:** 10.1186/s13063-026-09496-8

**Published:** 2026-01-30

**Authors:** Martin Alfuth, Anna Boehm, Jonas Klemp, Ryoko Kobayashi, Cathrin Bauer

**Affiliations:** Therapeutic Sciences, Faculty of Health Care, Niederrhein University of Applied Sciences, Reinarzstr. 49, Krefeld, 47805 Germany

**Keywords:** Ankle instability, Foot exercises, Function, Gait

## Abstract

**Background:**

The forefoot and rearfoot can individually contribute to the development of chronic ankle instability (CAI) after an ankle injury. The aim of this study is to evaluate the effects of targeted forefoot and rearfoot stability training on dynamic balance, postural stability, gait biomechanics, and self-reported joint function in individuals with CAI.

**Methods:**

This study is a prospective, single-center, interventional, randomized controlled trial with two comparison groups, either a usual balance training group or a control group. Individuals (18–44 years) with a self-reported ankle instability, a history of at least one ankle injury at least 12 months ago with typical signs of inflammation, two or more episodes of “giving way” within the previous 6 months, and a Cumberland Ankle Instability Tool score ≤ 24 are included. Exclusion criteria include acute injury, lower extremity surgery or fracture, neurological disease, chronic overuse injuries such as Achilles or patellar tendinopathy, and current participation in a targeted ankle rehabilitation program. The primary outcome measures are dynamic balance as measured by the Y-Balance Test and postural stability as assessed by the Modified Balance Error Scoring System. Secondary outcome measures include gait biomechanics measured by 3D gait analysis and self-reported ankle function assessed by the Foot and Ankle Ability Measure.

**Discussion:**

Since there is a lack of information regarding the effects of targeted forefoot and rearfoot exercises on ankle stability, this study has the potential to improve future treatment plans and provide healthcare practitioners with an alternative treatment for CAI.

**Trial registration:**

DRKS (German Clinical Trials Register)—DRKS00034295, retrospectively registered on May 22, 2024 (Reason: The study was originally designed as a randomized controlled pilot study; therefore, the first participants were enrolled without registering the study, which was then caught up as soon as possible); URL: https://drks.de/register/de/trial/DRKS00034295/preview.

## Introduction

### Background and rationale {9a}

With an incidence of 1:10,000 people per day, a lateral ligament injury is the most common sports injury. Lateral ligament injuries can also occur during everyday activities [1]. For basketball players, it has been found that approximately 90% of the injured players are under the age of 40, and more men than women suffer from ligament injuries [2]. Approximately 32–74% of individuals experience recurrent symptoms such as reinjury or persistent ankle instability [3, 4]. In 40–70% of cases, chronic ankle instability (CAI) is the result of an injury to the capsule-lateral ligament apparatus [5].

CAI is characterized by a history of ankle injury with typical signs of inflammation, a “giving way” or feeling of instability, and relevant limitations in the patient’s daily activities [6]. In addition, using the Short-Form-36 questionnaire (SF-36), it has been found that individuals with CAI have a significantly reduced quality of life compared to those without CAI. Biomechanical studies have shown deficits in gait and postural control/balance [7–9]. In the first half of the stance phase, there is an increased rearfoot inversion angle, which results in more rolling over the lateral border of the plantar foot. This leads to increased stress in this area and enhances the risk of a recurrent ankle sprain [7, 10, 11]. The altered foot rollover also affects knee joint kinetics in the early gait cycle, i.e., increased knee abduction moment, as well as ankle joint kinetics and muscle activity in the late gait cycle, i.e., increased eversion moment and peroneal muscle activity to compensate for the maladaptive gait pattern [7].

The interaction between rearfoot and forefoot individually contributes to the development of chronic ankle instability following an ankle injury [12]. A variety of joint movements can be observed in participants with chronic ankle instability:

- Increased eversion and joint play during internal talocrural rotation of the rearfoot
- Increased flexion motion of the forefoot relative to the rearfoot and first metatarsophalangeal joint
- Hypomobility of forefoot inversion

Fraser et al. observed increased rearfoot inversion 2 weeks after a lateral ankle sprain (LAS) [12]. This modified movement following an LAS can be considered a preliminary CAI [13]. Furthermore, increased forefoot eversion has been shown to be associated with greater laxity in the metatarsal joints and the fibularis longus muscle [14]. The interaction of altered biomechanics and a lack of therapeutic and/or medical intervention following LAS has been found to result in CAI [15].

During normal slow walking, foot lengthening flattens the medial arch to improve ground contact and balance control [16]. During fast walking, foot lengthening in the stance phase is reduced due to increased longitudinal arch stiffness to improve pushing off the foot by optimizing the lever arm. The symptoms described above can disrupt these normal foot biomechanics during gait and promote functional deficits in patients with CAI. Deficits in patients with CAI have been identified using magnetic resonance imaging of the muscles in the affected leg and foot. Both the muscles of the posterior superficial compartment and the small foot muscles have reduced muscle volume compared to healthy participants [17].

Non-surgical treatment is recommended for CAI [18]. Standard care includes balance training, ankle strengthening, and flexibility exercises. Training approaches for postural stability are stabilization exercises [18, 19]. These should be performed first on a flat surface, then on an unstable surface such as the wobble board, and then with additional coordinative-cognitive demands such as closed eyes. Gait training is also recommended [18]. The standard treatment, balance training with a wobble board, and therefore the comparator in this study, is limited by the inability to specifically control the numerous articulations between the rearfoot, midfoot, and forefoot that are important for the functional coupling of the forefoot and rearfoot. This is necessary to prevent potential sequelae such as the development of osteoarthritis [15]. The limitations of balance training described above may be overcome by supplementing it with targeted forefoot and rearfoot training. There is still a lack of information on the effects of targeted forefoot and rearfoot training to improve ankle function. Two studies have been conducted that compared lower extremity muscle activity while standing on various unstable training devices [20, 21]. Additionally, one single-case experimental study examined the effects of targeted rearfoot and forefoot training on dynamic balance and joint function in three soccer players [22].

In the study by Sánchez-Barbadora et al. [20], the muscle activity of lower extremity muscles was measured in 20 healthy active subjects using various balance training devices (BOSU, wobble board, power board, and Blackboard). The balance training devices were divided into two categories: global balance devices, such as the BOSU, wobble board, power board, and a balance training device that distinguishes between the forefoot and rearfoot, such as the Blackboard. Surface electromyography (sEMG) was used to record the activity of the peroneus longus (PL), peroneus brevis (PB), soleus (SOL), gastrocnemius medialis (GM), gastrocnemius lateralis (GL), and tibialis anterior (TA) muscles. All tests were carried out in a standing position and in a single-leg squat position on each balance training device. The Blackboard was only tested with an unstable rearfoot position. All tests showed similar activation of the PL muscle on each device. For all other muscles, the activation for standing or squatting on the Blackboard was lower than for the devices designed for global instability.

In another study, Alfuth and Gomoll [21] examined the muscle activity of the lower extremities of 27 healthy adults under one stable and five unstable conditions. The Mini Stability Trainer (ARTZT vitality®, Ludwig ARTZT GmbH, Dornburg, Germany) was used for four of the unstable conditions with various modifications, and the Balance Pad (BP) was used for one of the unstable conditions. Surface electromyography (sEMG) was used to record the activity of the TA, PL, SOL, GM, long head of the biceps femoris (BF), and vastus medialis (VM) muscles. The TA, PL, and BF muscles demonstrated significantly higher mean activity under the BP condition and the forefoot unstable in the frontal plane and multidirectional conditions compared with the control condition. The PL muscle exhibited significantly higher mean activity under the rearfoot unstable in the frontal plane condition. The SOL exhibited significantly higher levels of activity when balancing on the BP. The VM muscle exhibited significantly higher activity levels under conditions involving forefoot instability in the frontal plane and multidirectional movement.

In a single-case experimental study conducted by Alfuth and Schroers [22], three soccer players participated in a 6-week training program using the Mini Stability Trainer. The participants were divided into two groups: one that began with forefoot training and one that began with rearfoot training. The training modalities were changed after 3 weeks. Measurements were taken before the training program began, after 3 weeks, and after an additional 3 weeks. The two subjects who started with forefoot training exhibited no changes in the Cumberland Ankle Instability Tool (CAIT) after 3 weeks. After 6 weeks and transitioning to rearfoot training, the players demonstrated improvements of approximately 1 and 5 points, respectively. The third participant began the program with rearfoot training and subsequently improved by three points on the CAIT after 3 weeks. After transitioning to forefoot training and completing an additional 3 weeks, they demonstrated a 2-point increase in CAIT scores. All players demonstrated an enhancement in Foot and Ankle Outcome Score values following a 3-week period, with an additional enhancement after 6 weeks. In the YBT, the players demonstrated marginal improvements.

### Explanation for the choice of comparator {9b}

The study comprises three comparison groups. The initial intervention is constituted by training with the Mini Stability Trainer. The Mini Stability Trainer facilitates targeted training of the forefoot and rearfoot. The targeted joints are of particular importance for the functional coupling of the forefoot and rearfoot. This is necessary to prevent possible consequences such as the development of osteoarthritis [15]. The second group is the standard treatment, which consists of balance training with a Wobble Smart Board. The primary objective of the study is to determine the comparative efficacy of the novel training intervention employing the Mini Stability Trainer in comparison to the conventional standard treatment. The second comparison is a control group without any training to assess if training with the Mini Stability Trainer is more effective than no training at all.

### Objectives {10}

The study will be guided by the following specific objective: To determine whether the outcomes of dynamic balance, postural stability, gait biomechanics, and joint function improve in the intervention group and the balance training group after a 6-week intervention period and whether there is a significant difference compared to the control group.

The study is also based on the following hypotheses:

1. The intervention group and the balance training group will lead to similar significant improvements in outcomes compared to the control group.
2. It is also hypothesized that there will be no significant differences between the intervention group and the balance training group.

## Methods: patient and public involvement, and trial design

### Patient and public involvement {11}

Patients are included in the study to complete the 6-week study phase, as well as the preliminary and post-examination and follow-up. If members of the training groups are using the Mini Stability Trainer or the Wobble Smart Board, it is imperative that they complete the training sessions during the study phase. The study also includes members of the public, students, physiotherapists, and various sports groups to establish contact between patients and study employees.

### Trial design {12}

This study protocol follows the “Standard Protocol Items: Recommendations for Interventional Trials (SPIRIT) 2013 Statement” (https://www.equator-network.org/reporting-guidelines/spirit-2013-statement-defining-standard-protocol-items-for-clinical-trials/). The study is a prospective, single-center, interventional, randomized controlled trial with an intervention group (forefoot and rearfoot stability training) and two comparison groups, either a usual balance training group or a control group. It is a single-blind (principal investigator) design with patients randomized 1:1:1 to one of the three parallel groups. To ensure internal validity, a control group is included that does not receive any balance training intervention. Because many patients with CAI do not seek medical or physical therapy and continue with their usual activities, participants in the control group are allowed to do so. A summary of study information is presented in Table 1.

**Table 1:** Study information

Category	Information
Country	Germany
Intervention	Active part: 6 weeks of training with the Mini Stability Trainer according to a predefined exercise schedule; 6 weeks of training with the Wobble Smart Board according to a predefined exercise schedule; one training session is performed under the supervision of a member of the study team, at least 17 training sessions are performed independently by each subject at home Control group: activities of daily living and sports activities; balance exercises are not allowed
Inclusion and exclusion criteria	Inclusion criteria: age between 18 and 44 years; based on the guidelines of the International Ankle Consortium: history of at least one ankle injury more than 1 year ago with typical signs of inflammation; self-reported ankle instability; in addition, two or more episodes of “giving way” must have occurred within the previous 6 months; Cumberland Ankle Instability Tool (CAIT ^a^) score ≤ 24 (in the case of bilateral ankle instability, the more severely affected side is selected) Exclusion criteria: acute injury, surgery or fracture of the lower extremity, neurological disorders, chronic overuse injuries such as Achilles or patellar tendinopathy; current participation in a targeted ankle rehabilitation program
Study type	Allocation: randomized; model: parallel allocation; intervention description: comparison of clinical outcome measures at four time points between three cohorts (Wobble Smart Board, Mini Stability Trainer, control group) of subjects with chronic ankle instability (CAI ^b^), whether the two intervention groups reduce the primary and secondary outcome measures compared to the control group; blinding: single-blind; blinding description: the principal investigator analyzing the results will be blinded to group allocation; different investigators will be used to perform the intervention and to analyze the results
Primary outcomes	Dynamic balance and postural stability using the Y-Balance Test (YBT ^c^) and Modified Balance Error Scoring System (MBESS d)
Secondary outcomes	Gait biomechanics using 3D gait analysis (e.g., ankle abduction and adduction; knee varus/valgus position; spatiotemporal parameters); ankle function using the Foot and Ankle Ability Measure (FAAM ^e^)

^a^*CAIT *Cumberland Ankle Instability Tool; ^b^*CAI *chronic ankle instability; ^c^*YBT *Y-Balance test; ^d^*MBESS *Modified Balance Error Scoring System; ^e^*FAAM *Foot and Ankle Ability Measure

## Methods: participants, interventions, and outcomes

### Trial setting {13}

The present study is being conducted at the Movement Laboratory of the Faculty of Health Care at Niederrhein University of Applied Sciences in Krefeld, Germany. The Movement Laboratory is equipped with a 10-m-long walkway with two integrated force plates (Bertec, version 1.0.0., Bertec Corporation, Columbus, OH, USA). A motion analysis system with 10 cameras (Qualisys, Göteborg, Sweden) is also available for motion analysis. Characteristics of the people who are needed for the trial are presented in Table 2.

**Table 2:** Characteristics of the people who are needed for the trial

Characteristic	The people we would expect to see included
Age	18 to 44 years
Sex	Male, female, and intersex
Gender	Man and woman together with any other gender identities
Race, ethnicity, and ancestry	Patients with CAI from all races, ethnicities, and ancestries are eligible to participate in the study
Socioeconomic status	Patients with CAI from all socioeconomic status are eligible to participate in the study
Geographic location	Patients with CAI from all geographic locations are eligible to participate in the study, but have to carry out the preliminary examination and post-examination in the Movement Laboratory
Other characteristics relevant to the trial	Not applicable

### Eligibility criteria for participants {14a}

To be eligible for the study, patients must meet the following predefined inclusion criteria:

- Age between 18 and 44 years
- History of at least one ankle injury ≥ 12 months ago with typical signs of inflammation
- Self-reported ankle instability
- Two or more episodes of “giving way” within the previous 6 months
- Cumberland Ankle Instability Tool (CAIT) score ≤ 24 (in the case of bilateral ankle instability, the more severely affected side is selected)

Exclusion criteria are the following:

- Acute injury
- Lower extremity surgery or fracture
- Neurological disease
- Chronic overuse injuries such as Achilles or patellar tendinopathy
- Currently participating in a targeted ankle rehabilitation program

### Eligibility criteria for sites and those delivering interventions {14b}

No additional centers or intervention providers are required for the study. The preliminary examination and post-examination are conducted exclusively in the Movement Laboratory of the Faculty of Health Care at Niederrhein University of Applied Sciences in Krefeld, Germany.

### Who will take informed consent? {32a}

The physiotherapist AB obtains the informed consent before the preliminary examination.

### Additional consent provisions for collection and use of participant data and biological specimens {32b}

Additional consents for the collection and use of participant data are included in the informed consent form and are obtained by AB prior to the preliminary examination. No biological samples are used in this study.

## Intervention and comparator

### Intervention and comparator description {15a}

In total, both intervention groups perform three training sessions per week independently at home for 6 weeks. After the initial measurement, a supervised training session is conducted in the laboratory by a state-certified physiotherapist (AB) to instruct and familiarize the participant with the exercises. The participant then performs a total of 17 unsupervised training sessions at home, following a detailed training schedule according to their group assignment (Tables 3, 4, and 5). Training is gradually increased every 2 weeks to induce improvements in performance and function. The training devices, i.e., Mini Stability Trainer (ARTZT vitality®, Ludwig ARTZT GmbH, Dornburg, Germany) or Wobble Smart Board (ARTZT vitality®, Ludwig ARTZT GmbH, Dornburg, Germany), and the training schedule are given to the participants by the investigator after the supervised training session. The Mini Stability Trainer can stabilize or destabilize the rearfoot and forefoot separately. It has four small, flat plates with different tilting options: two straight lines for stable standing, a center line for tilting right and left, and a semicircle for tilting in all directions. To target rearfoot or forefoot stabilization, one plate is placed under the forefoot and the other under the rearfoot. The Wobble Smart Board is a round plate with an adjustable fulcrum that stabilizes the entire ankle-foot complex. Detailed information about the training can be found in Tables 3, 4, and 5. The characteristics of the training equipment and its effect on muscle activity and postural control have been described previously [21–23]. There are six exercise variants for training with the Mini Stability Trainer and the Wobble Smart Board (Tables 3, 4, and 5). The exercises are divided into three blocks, so that two exercises are performed for 2 weeks each. The exercise parameters, including duration, intensity, and frequency, are based on reported sensorimotor training [24] and are presented in Tables 3, 4, and 5.

**Table 3:** Schedule for the two exercise groups (Mini Stability Trainer; Wobble Smart Board) for the first 2 weeks

Mini Stability Trainer	Wobble Smart Board
For each training week 3 times per week with a minimum of 1 day and a maximum of 2 days rest in between	
Weeks 1 + 2	
Exercise 1	
• Place hands on iliac crest • Step the affected foot forward on the Mini Stability Trainer (forefoot on the green plate/rearfoot on the blue or yellow plate with tilt to the left and right) • Look forward • Shift weight evenly to front foot with knee slightly flexed and hold for 20 s (opposite foot maintains light contact with floor with big toe) • Rest for 30 s • Repeat with opposite foot • Repeat 5 times for each side	• Place hands on iliac crests • Step forward with the affected foot onto the center of the Wobble Smart Board (difficulty level according to your own ability) • Look forward • Shift weight evenly to front foot with knee slightly flexed and hold position for 20 s (opposite foot maintains light contact with floor with big toe) • Rest for 30 s • Repeat with opposite foot • Repeat 5 times for each side
Two-minute rest after exercise 1 Exercise 2	
• Place hands on iliac crest • Step with the affected foot to the side on the Mini Stability Trainer (forefoot on the green plate/rearfoot on the red plate with inclination in all directions) • Look forward • Shift weight evenly to affected foot with knee slightly flexed and hold position for 20 s (opposite foot maintains light contact with floor with big toe) • Rest for 30 s • Repeat with opposite foot • Repeat 5 times for each side	• Place hands on iliac crest • Step with the affected foot from the side to the center of the Wobble Smart Board (difficulty level according to your own ability) • Look forward • Shift weight evenly to affected foot with knee slightly flexed and hold position for 20 s (opposite foot maintains light contact with floor with big toe) • Rest for 30 s • Repeat with opposite foot • Repeat 5 times for each side

**Table 4:** Schedule for the two exercise groups (Mini Stability Trainer; Wobble Smart Board) for weeks 3 and 4

Mini Stability Trainer	Wobble Smart Board
For each week of training 3 × per week with a minimum of 1 day and a maximum of 2 days rest in between	
Weeks 3 + 4	
Exercise 3	
• Place hands on iliac crest • Step forward with affected foot on Mini Stability Trainer in one-legged stance with knee slightly flexed (forefoot on the blue or yellow plate with inclines to left and right/rear foot on the green plate) • Look forward • Hold position for 25 s (opposite foot off the floor) • Rest for 30 s • Repeat with opposite leg • 5 times each side total	• Place hands on iliac crest • Step forward with the affected foot onto the center of the Wobble Smart Board in a one-legged stance with the knee slightly flexed (increase the difficulty by 1 from the first 2 weeks of training) • Look forward • Hold position for 25 s (opposite foot off the floor) • Rest for 30 s • Repeat with the opposite leg • 5 times each side total
Two-minute rest after exercise 3 Exercise 4	
• Place hands on iliac crest • Step with the affected foot to the side on the Mini Stability Trainer in a one-legged stance with the knee slightly flexed (forefoot on the red plate with tilt in all directions/rearfoot on the green plate) • Look forward • Hold position for 25 s (opposite foot off the floor) • Rest for 30 s • Repeat with opposite leg • 5 times each side total	• Place your hands on pelvic crest • Step the affected foot from the side to the center of the Wobble Smart Board in a one-legged stance with the knee slightly bent (increase the difficulty by 1 compared to the first 2 weeks of training) • Look forward • Hold position for 25 s (opposite foot off the floor) • Rest for 30 s • Repeat with opposite leg • Repeat 5 times for each side

**Table 5:** Schedule for the two exercise groups (Mini Stability Trainer; Wobble Smart Board) for the last 2 weeks

Mini Stability Trainer	Wobble Smart Board
The following applies for each training week: 3 × per week with at least one day and a maximum of 2 days rest in between	
Weeks 5 + 6	
Exercise 5	
• Place hands on iliac crest • Step forward on the Mini Stability Trainer with the affected foot in a one-legged stance with the knee slightly flexed (forefoot on the red plate with tilt in all directions/rearfoot on the green plate) • Look forward • Hold position for 30 s (opposite foot off the floor) • Rest for 30 s • Repeat with opposite leg • 5 times each side total	• Place hands on iliac crest • Step forward with the affected foot to the center of the Wobble Smart Board in a one-legged stance with the knee slightly flexed (increase difficulty by 1 compared to both training weeks 3 + 4) • Look forward • Hold position for 30 s (opposite foot off the floor) • Rest for 30 s • Repeat with opposite leg • 5 times each side total
Two-minute rest after exercise 5 Exercise 6	
• Place hands on iliac crest • Step with affected foot to the side on the Mini Stability Trainer in one-legged stance with knee slightly flexed (forefoot on the red plate with tilt in all directions/rearfoot on the green plate) • Look forward • Hold this position for 30 s (opposite foot off the floor) and tap the floor alternately with the opposite foot forward, to the side, and backward • Rest for 30 s • Repeat with opposite leg • Repeat 5 times for each side	• Place hands on iliac crest • Step with the affected foot from the side to the center of the Wobble Smart Board into a one-legged stance with the knee slightly flexed (increase the difficulty by 1 compared to both training weeks 3 + 4) • Look forward • Hold this position for 30 s (opposite foot off the floor) and tap the floor alternately with the opposite foot forward, to the side, and backward • Rest for 30 s • Repeat with opposite leg • 5 times each side total

Participants may withdraw from the study at any time without giving a reason. If patients are unable to perform a test during laboratory measurements or home exercise, or if they need to deviate from the prescribed exercise plan, this will be documented and taken into account in data analysis and interpretation. If a participant is required to receive the other group’s treatment or to switch to the control group (e.g., experiencing significant pain during exercise), or if values are missing for any reason (e.g., dropout or loss to follow-up), this is accounted for using intention-to-treat (ITT) analysis.

### Criteria for discontinuing or modifying allocated intervention/comparator {15b}

The criteria for terminating or modifying the intervention or comparison are pain with signs of inflammation during or after the training sessions, as well as illnesses that prevent training.

### Strategies to improve adherence to intervention/comparator {15c}

After randomization to one of the two intervention groups, the participants receive the appropriate training device for the respective intervention with detailed training instructions from the physiotherapist (AB). In addition, they receive a training booklet with all exercise modalities (Tables 3, 4, and 5). As previously mentioned, a supervised training session is conducted in the laboratory by the physiotherapist to instruct and familiarize the participant with the exercises. To ensure adherence with the home training, each participant is instructed to check a box in the training booklet after each session. Participants are also asked to document any peculiarities, such as pain or other symptoms, that may have occurred during the exercise. During the study, participants can contact the physiotherapist at any time via email or once a week via Zoom to clarify any questions they may have. Each participant gets financial compensation (20 €) after the second laboratory measurement after 6 weeks. Participants are informed that they may engage in their usual activities, including sports, but that additional balance or sensorimotor training is not permitted during the trial, including follow-up. Participant insurance is provided to all study participants. However, there is no provision for post-trial care.

### Concomitant care permitted or prohibited during the trial {15d}

Due to CAI, treatments such as physiotherapy, medication, and drug therapy are not permitted. Special balance training for the ankle is also prohibited. Participants may engage in all activities of daily living (ADL) and sports activities, including training sessions.

### Ancillary and post-trial care {34}

Participants have the opportunity to liaise with physiotherapist AB via email during and after the program. Additionally, the program incorporates weekly meetings that can be conducted via Zoom.

### Outcomes {16}

#### Primary outcome measurements

##### Y-Balance Test

The Y-Balance Test (YBT) is a test of dynamic balance and is administered using a standardized test kit [25, 26]. In this study, the participant stands barefoot with the test leg on the stance platform of the test kit with the most distal portion of the big toe at the starting line. The hands are placed on the left and right iliac crest. The participant is instructed to move the target (reach indicator) with the free leg along the pipe as far as possible in the anterior, posteromedial, and posterolateral directions and to hold the end position for 3 s. Six trials are performed in each direction [27]. The first 3 trials are used to familiarize the participant with the task. The following 3 trials are recorded from the tape measure (5 mm increments) of each pipe at the edge of the reach indicator, at the point where the most distal point of the foot reaches, and used for further analysis. The participant is instructed to maintain balance during the trials and to return to the starting position after each trial. A trial is considered invalid and will be repeated if the participant touches the ground with the free leg, slips with the standing foot, falls off the stance platform, loses contact with the reach indicator while moving it to the maximum reach position, kicks the reach indicator forward, places the foot on the top of the reach indicator, or fails to return to the starting position under control. The maximum number of attempts is 3. The left leg is measured first. In addition, leg length is measured from the anterior superior iliac spine to the medial malleolus with the participant in the supine position to normalize anterior, posteromedial, and posterolateral reach distances to leg length. The following formula is used for normalization: reach distance/leg length × 100. The means of these normalized values are then calculated. In addition, the composite score is calculated using the following formula: (sum of the 3 reach distances)/3 times leg length × 100. In this study, the results are presented using the composite score. The minimum detectable change in YBT is between 7.7 and 14% [28]. The difference in change between the groups is assessed and calculated using parametric statistical analysis. The aggregation method is mean ± SD, and the measurements are taken before and after the 6-week intervention.

##### Modified Balance Error Scoring System

In this study, the Modified Balance Error Scoring System (MBESS), which measures static balance, is performed on a foam surface (Balance Pad, ARTZT vitality®, Ludwig ARTZT GmbH, Dornburg, Germany) [29]. To perform the test, the participant stands in three positions on the balance pad with hands on the left and right iliac crest, eyes closed, and attempts to maintain the position for 20 s. Balancing in each of the three stance positions is scored out of a maximum of 10 points each. Errors are subtracted from the score, resulting in a maximum total MBESS score of 30 [30]. Errors are scored if the eyes are opened, the hands are removed from the pelvis, a step, trip, or fall occurs, hip abduction or flexion exceeds 30°, or the forefoot or heel is lifted. If the participant can hold the position for less than 5 s, the position is penalized with ten penalty points [31]. Reliability of the MBESS is moderate to strong with coefficients ranging from r = 0.6–0.88 [30]. The difference in change between groups is assessed and calculated using nonparametric statistical analysis. The aggregation method is median and interquartile range, and measurements are taken before and after the 6-week intervention.

#### Secondary outcome measurements

##### Function of the ankle joint

The German version of the Foot and Ankle Ability Measure (FAAM) questionnaire is a validated patient-reported outcome measure to identify functional limitations in individuals with CAI [32–34]. Twenty-one items relate to the Activities of Daily Living (ADL) subscale (FAAM-ADL) and eight items relate to the Sport subscale (FAAM-Sport) [35]. For each item, the possible responses range from 0 “no problems” to 4 “unable to do anything.” The score for each item in each subscale is summed (maximum score for the ADL subscale = 84; maximum score for the Sport subscale = 32). The sum is divided by the maximum score and then multiplied by 100. A resulting maximum score of 100 represents no functional limitation in each subscale. In patients with chronic ankle instability, FAAM-ADL and FAAM-Sport scores of <90 and <80, respectively, are considered to represent functional disability. The questionnaire is also suitable for recording changes over time. The minimum detectable change (MDC) is ±5.7 points for the ADL subscale and ±12.3 points for the Sport subscale [35]. The difference in change between groups is assessed and calculated using nonparametric statistical analysis. The aggregation method is median and interquartile range, and measurements are taken before and after the 6-week intervention, as well as at the 4- and 12-week follow-ups.

##### Gait biomechanics

In order to assess the gait biomechanics, a 3D gait analysis is performed in the Movement Laboratory of the Niederrhein University of Applied Sciences using two force plates (Bertec, Columbus, OH, USA) with a sampling frequency of 1000 Hz, including a low-pass filter of 500 Hz, and a 10-camera motion analysis system (Qualisys, Göteborg, Sweden), which captures kinematic data at 340 Hz. The two force plates are integrated into a 10-m-long walkway and are later used to detect touch down and toe off events of the foot during gait. Prior to the gait analysis, the measurement system is calibrated according to the manufacturer’s specifications. Then, 26 retro-reflective markers according to the Istituti Ortopedici Rizzoli (IOR) lower body marker set are placed on bony reference points with double-sided tape to record the participants’ gait kinematics [36]. The IOR marker set for the lower body is chosen over other specific marker sets, such as the Oxford foot model and the modified Rizzoli foot model, because Qualisys recommends it and it is well-established in clinical settings. This study focuses on assessing general spatiotemporal gait parameters and the kinematics of major lower extremity joints relevant to activities of daily living and sports, rather than fine movements between the forefoot and rearfoot. The latter may be of interest when specific questions arise regarding ankle and foot joint movements. A static reference measurement is taken from each participant, which is later used for scaling purposes. Participants are then instructed to walk across the walkway at their self-selected walking speed and five valid right and left trials are recorded. Kinematic and kinetic data are processed using QTM Qualisys Track Manager—software, version 2022.2 (Qualisys, Göteborg, Sweden). Lower extremity joint angles, ground reaction forces, joint moments, and spatiotemporal gait parameters are presented in a gait analysis report based on a template by Baker et al. [37]. Relevant gait parameters are vertical, antero-posterior and mediolateral ground reaction forces (N), ankle, knee and hip angles (°), and cadence (steps/min), step length (m), stride length (m), stride width (m), and gait speed (ms−1). In addition, data are processed using the visual 3D software (©HAS-Motion, Canada). Instrumented gait analysis is considered reliable, with mean intraclass correlation coefficients (ICC) ranging from 0.95 to 0.97, mean standard error of measurement (SEM) values ranging from 2.01 to 2.47 degrees, and mean MDC values ranging from 5.86 to 7.02 degrees, and represents the gold standard for gait assessment in individuals with gait abnormalities [38, 39]. For all gait parameters, the difference in change between the groups is assessed and calculated using parametric statistical analysis. The method of aggregation is mean ± SD, and the measurements are taken before and after the 6-week intervention.

### Harms {17}

Adverse events, such as a recurrent ankle injury, other acute injuries, complaints, or illnesses, and any identifiable harm that the participant may experience as a result of the intervention, such as physical or psychological symptoms, are documented promptly. For this purpose, the investigator maintains regular contact with the participants. All adverse events will be reported in future publications. Participants are asked to report any significant deterioration of the ankle joint to the investigator immediately so that any necessary examinations can be initiated. Participant insurance is provided to all study participants. However, there is no provision for post-trial care.

### Participant timeline {18}

After a potential participant has contacted the study staff (AB) via email, the participant receives a self-administered questionnaire asking for basic socio-demographic and injury-related data and the CAIT to complete the initial eligibility screening. If the participant meets the eligibility criteria, an appointment is made for the first measurement in the laboratory. At the first appointment, all eligibility criteria are finalized and the participant is enrolled after providing informed consent. Outcome measures are then completed and the participant is assigned to one of the three groups by closed envelope. At the second appointment after the 6-week intervention, outcome measures are repeated. At the 4-week and 12-week follow-up visits, participants are contacted by email and asked to complete the FAAM questionnaire. A detailed description of the study procedure can be found in Table 6.

**Table 6:** Study procedure

Time point	Investigation period				
	Entry	Baseline	According to the group categorization		
	<2 weeks	Time = 0 or preliminary examination and group classification	Intervention (6 weeks)	After the intervention	4 and 12 weeks after the intervention
Entry: Questionnaires	x				
Assignment		x			
Intervention					
Group 1: control			x		
Group 2: Mini Stability Trainer			x		
Group 3: Wobble Smart Board			x		
Assessments					
CAIT ^a^		x			
FAAM ^b^		x		x	x
YBT ^c^		x		x	
MBESS ^d^		x		x	
3D-Gait analysis		x		x	

^a^CAIT, Cumberland Ankle Instability Tool, ^b^ *FAAM *Foot and Ankle Ability Measure, ^c^*YBT *Y-Balance Test, ^d^*MBESS *Modified Balance Error System

### Sample size {19}

An a priori sample size estimation based on the results of pilot measurements [40] examining change in dynamic balance after 6 weeks of balance training using the Mini Stability Trainer indicated that 33 participants (11 per group) would be needed to detect a mean difference of 4.6% in the composite score of the YBT with an effect size of *f* = 0.33 and a probability of 1 − *β* = 0.9 at a significance level of *α* = 0.05. To account for potential dropouts and loss to follow-up, the target number of participants is 36 (12 per group).

### Recruitment {20}

A total of 36 male, female, and diverse individuals with CAI are recruited in and around Krefeld, Germany. Recruitment is done through flyers posted at the university and cooperation partners, social media postings, and word of mouth. In addition, sports clubs and groups of different sports (e.g., soccer, handball, basketball, tennis, running), rehabilitation centers, physiotherapy and medical practices, and hospitals are contacted via email and personal visits to inform them about the study.

## Assignment of interventions: randomization

### Sequence generation: who will generate the sequence {21a}

Sequence generation is performed by physiotherapist AB with the two student assistants RK and CB. Permuted block randomization is performed using the program DatInf RandList version 1.5 (Tübingen, Germany).

### Sequence generation: type of randomization {21b}

A permuted block randomization with blocks of 6 is performed using the program DatInf RandList version 1.5 (Tübingen, Germany). Participants are randomly assigned to either the control, Mini Stability Trainer (intervention), or Wobble Smart Board (comparison) groups with an allocation ratio of 1:1:1 (12 for control, 12 for Mini Stability Trainer, and 12 for Wobble Smart Board groups). Assignment of interventions is completed after the initial measurement using sequentially numbered, opaque, sealed envelopes to ensure no interference with group assignment. One physiotherapist (AB) and two occupational therapists (RK, CB) independently generate the allocation sequence, enroll participants, and assign participants to interventions.

### Allocation concealment mechanism {22}

Once randomization is complete, the group assignments are placed in opaque, sealed envelopes. The envelopes are numbered and opened exclusively by the respective participant to ensure blinding until allocation.

### Implementation {23}

The physiotherapist AB and the principal investigator MA have access to the random allocation sequence. Access to the file is dependent on the verification of the file with the user’s personal password.

## Assignment of interventions: blinding

### Who will be blinded {24a}

Since the interventions are performed by the participants, they are visible to them. Therefore, it is not possible to blind participants to the intervention. However, participants in the intervention groups are not told which intervention is being studied and which is the comparison. It is not possible to blind participants in the control group. The observer performing the measurements assigns participants to groups using sealed envelopes after the baseline measurements and is therefore blinded to group assignment at baseline but is not blinded to group assignment at post-measurement and follow-up. In this way, participants are also blinded and uninfluenced by the group assignment at baseline, and possible biases or expectations will not affect the results. The principal investigator who analyzes the data is blinded to group allocation throughout the study.

### How will be blinding be achieved {24b}

The blinded principal investigator is unaware of the group allocation of participants because the envelopes are sealed and inaccessible to him.

### Procedure for unblinding if needed {24c}

Not applicable; the participants in the study are not blinded with regard to their intervention.

## Data collection and management

### Plans for assessment and collection of outcomes {25a}

A detailed description of the outcome assessments and measurement instruments, including information on reliability, validity, SEM, and MDC, is provided in the outcome section. All assessments and measurements were trained by the assessor prior to conducting the study until full proficiency was achieved. The German versions of the CAIT and the FAAM are completed by the participants using prepared paper sheets [34, 41]. The results are then converted into prepared Word documents by the assessor. The YBT and MBESS scores are documented by the assessor immediately after the measurement using a priori constructed Word documents. Criteria for discontinuing or modifying allocated interventions are already mentioned in item 15a, and strategies for promoting participant adherence to each intervention are already mentioned in item 15c.

### Plans to promote participant retention and complete follow-up {25b}

In order to motivate participants during the 6-week study period, a participant fee of €20 will be paid after the post-examination. Furthermore, all participants will be provided with a comparison of their results, accompanied by explanatory notes, upon completion of the 12-week follow-up. The control group will receive the training plans for both groups after completing the 12-week follow-up.

### Data management {26}

Participants’ demographic and injury-related data are collected using a self-administered questionnaire. CAIT and FAAM data are documented using predesigned paper sheets. YBT and MBESS scores are documented by the assessor immediately after each measurement using a predesigned electronic Word document. Data are later exported to Excel files (Microsoft Excel Professional Plus 2019, Redmond, USA) for further processing. Gait analysis data from the respective measurement software are exported individually into Excel files (Microsoft Excel Professional Plus 2019, Redmond, USA) immediately after data collection. All relevant data are also transferred to a prepared Excel master spreadsheet for further analysis. All data are stored on a password-protected computer and on a secure server within the university computer system. In addition, data are stored on a password-protected external hard drive. Printed paper forms are kept in separate files for 10 years after the study is closed. Only study staff have access to the study files and the folders in which the database is stored. All data are checked before being transferred to the statistical software for inferential analysis (IBM SPSS Statistics for Windows, version 29.0.2.0 Armonk, NY: IBM Corp). Data integrity is ensured by referential data and range checks by two separate therapists. An individual privacy policy regarding disclosure/data collection, data management, data labeling, and data protection is included in the ethics approval.

### Confidentiality {33}

Before, during, and after the study, data are pseudonymized with a unique identification number for each participant and stored in folders accessible only to study team members to ensure confidentiality. Any records that contain names or other personal identifying information are kept separately from those used to record the study. The computers used and the external hard drive are password-protected, and only study team members have access to them. The data is stored on the university’s server, to which only members of the study team have access.

## Statistical methods

### Statistical methods for primary and secondary outcomes {27a}

Primary and secondary outcome measures are reported descriptively using mean ± SD or median and interquartile range according to their type (e.g., ordinal or interval scale). After testing the data for normal distribution using the Shapiro-Wilk test and histograms, for the primary outcomes of dynamic balance (YBT) and postural stability (MBESS), the significance of differences in post-intervention change within and between groups will likely be analyzed using repeated measures analysis of variance (ANOVA) (YBT) or Friedman tests or Kruskal-Wallis tests (MBESS) and post hoc tests with Bonferroni correction to account for multiple testing. After testing the data for normal distribution using the Shapiro-Wilk test and histograms, for the secondary outcome of gait biomechanics, the significance of differences in post-intervention change within and between groups will likely be analyzed using repeated measures analysis of variance (ANOVA) and post hoc tests with Bonferroni correction to account for multiple testing. For the secondary outcome of joint function (FAAM), the significance of differences in post-intervention change within and between groups will be analyzed using Friedman tests or Kruskal-Wallis tests and post hoc tests with Bonferroni correction to account for multiple testing. The global significance level is set to *α* = 0.05. No additional analyses are planned.

### Who will be included in each analysis {27b}

All randomized participants will be included in each analysis as randomized.

### How missing data will be handled in the analysis {27c}

If a participant is required to receive the other group’s treatment or switch to the control group (e.g., if they experience significant pain during exercise), or if values are missing for any reason (e.g., dropout or loss to follow-up), this is accounted for using ITT analysis. This means that participants are analyzed in the group to which they were randomized, and missing values are imputed. The imputation method (single or multiple imputation) is determined according to the guidelines of Jakobsen et al. [42] at the end of data collection.

### Methods for additional analyses (e.g., subgroup analyses) {27d}

No additional analyses (e.g., subgroup and adjusted analyses) are planned.

### Interim analyses {28b}

No interim analyses are planned.

### Protocol and statistical analysis plan {5}

DRKS (German Clinical Trials Register)—DRKS00034295, https://drks.de/register/de/trial/DRKS00034295/preview

## Oversight and monitoring

### Composition of the coordinating center and trial steering committee {3d}

Data will be monitored by the assessor (AB) immediately after recording and documentation. All data will be monitored and analyzed by the blinded principal investigator (MA). Participants will be seen regularly during the intervention to adjust the exercise protocol if necessary. As there is no risk of harm to participants, a formal data monitoring committee is not required for this study.

### Frequency and plans for auditing trial conduct {29}

Not applicable.

### Protocol amendments {31}

Any changes to the trial protocol will be communicated to the ethics committee and the trial registry.

### Dissemination policy {8}

We will share our research with academic and professional audiences by presenting at conferences and publishing our findings in high-impact journals.

## Discussion

The purpose of this study is to evaluate the effects of targeted forefoot and rearfoot stability training on the primary measures of dynamic stability and postural stability, as well as the secondary measures of gait biomechanics and self-reported function on the CAI. We hypothesize that the targeted forefoot and rearfoot group, as well as the wobble board group, will show significant improvements in the aforementioned measures but will not show significant differences between these intervention groups. Since there is currently a lack of information regarding targeted forefoot and rearfoot exercises on ankle stability, this study has the potential to improve long-term outcomes regarding CAI and provide an alternative to healthcare practitioners in treatment. The data from this study could therefore be used in the future to develop treatment plans for CAI.

## Trial status

Enrollment of the first participant began on July 26, 2023, and the trial protocol remains unchanged. Recruitment is expected to be completed by January 2026.

## Data Availability

The data are stored on the universitys server, to which only the study team has access. The data will not be shared with third parties and will be analyzed independently by the study team. The principal investigator will make the data available upon request.

## Abbreviations

ADL: Activities of daily living
ANOVA: Analysis of variance
CAI: Chronic ankle instability
CAIT: Cumberland Ankle Instability Tool
EMG: Electromyography
FAAM: Foot and Ankle Ability Measure
IOR: Istituti Ortopedici Rizzoli
ITT: Intention-to-treat
LAS: Lateral ankle sprain
MBESS: Modified Balance Error Scoring System
MDC: Minimum detectable change
SEM: Standard error of measurement
SF-36: Short-Form-36 questionnaire
YBT: Y-Balance Test
